# Preoperative phenotypic stratification of primary central nervous system lymphoma using multiparametric MRI-based radiomics: prediction of germinal center B-cell-like and double-expression status

**DOI:** 10.3389/fonc.2026.1822040

**Published:** 2026-06-29

**Authors:** Lingxu Chen, Xiaochen Wang, Sihui Wang, Tong Chen, Xuening Zhao, Ying Yan, Mengyuan Yuan, Shengjun Sun

**Affiliations:** 1 Department of Radiology, Beijing Tiantan Hospital, Capital Medical University, Beijing, China; 2 Department of Radiology, Beijing Neurosurgical Institute, Beijing, China

**Keywords:** del, germinal center, MRI, primary cns lymphoma, radiomics

## Abstract

**Background:**

Primary central nervous system lymphoma (PCNSL) exhibits substantial biological heterogeneity, particularly across immunohistochemical subtypes such as double-expression lymphoma (DEL) and germinal center B-cell-like (GCB) phenotypes. This study aimed to develop and evaluate multiparametric MRI-based radiomics models for preoperative prediction of DEL andGCB status in PCNSL.

**Methods:**

We retrospectively included 160 pathologically confirmed PCNSL patients. Multiparametric MRI sequences, including T2-weighted (T2WI), T2-Fluid-Attenuated Inversion Recovery (FLAIR), contrast-enhanced T1-weighted (T1CE), and apparent diffusion coefficient (ADC), were analyzed. Enhancing tumor core and peritumoral edema were automatically segmented using nnU-NetV2-based models, and radiomics features were extracted from both regions across all sequences. After reproducibility filtering, ComBat harmonization, and multistep feature selection performed exclusively within the training cohort, six machine-learning classifiers were trained and evaluated in held-out internal test sets. Model performance was assessed using ROC and decision curve analysis, and SHAP-based feature interpretation.

**Results:**

For DEL classification, 12 radiomic features were retained. The SVM classifier achieved the best test performance, with an area under the ROC curve (AUC) of 0.807 (95% CI, 0.649–0.936), accuracy of 0.730, sensitivity of 0.714, and specificity of 0.750. For GCB/non-GCB classification, seven radiomic features were used for model construction. The Random Forest classifier achieved the highest test performance, with an AUC of 0.897 (95% CI, 0.796–0.973), accuracy of 0.846, sensitivity of 0.792, and specificity of 0.893.

**Conclusions:**

Multiparametric MRI-based radiomics analysis demonstrated promising performance for noninvasive prediction of DEL and GCB/non-GCB phenotypes in PCNSL. These findings suggest that MRI-derived radiomic features may capture imaging correlates of biological heterogeneity and may support preoperative risk stratification and individualized treatment planning.

## 1 Background

Primary central nervous system lymphoma (PCNSL) is an extranodal non-Hodgkin’s lymphoma confined to the central nervous system. More than 90% of PCNSLs are diffuse large B-cell lymphomas (DLBCLs). According to the 2016 WHO Classification of Tumors of Haematopoietic and Lymphoid Tissues, DLBCLs with co-expression of MYC and B-cell lymphoma (BCL2) proteins are defined as double-expressor lymphomas (DELs) (1). Recent studies have suggested that double expression PCNSL (DE-PCNSL) is associated with inferior progression-free survival (PFS) and a higher risk of early disease progression or relapse than non-DEL disease (2, 3). Notably, this adverse association persists even in patients receiving high-dose methotrexate-based or intensive MATRix-based treatment, supporting DEL status as a clinically relevant marker for treatment-related risk stratification (4).

Cell-of-origin (COO) classification is the canonical molecular framework for DLBCL, separating tumors into germinal-center B-cell–like (GCB) and non-GCB subtypes (5, 6). Previous studies in systemic DLBCL have shown that the non-GCB subtype is associated with an adverse prognosis (7). However, in PCNSL, the prognostic value of GCB-type remains controversial; some studies reported longer survival (8, 9), while others found no significant association with overall survival (10). Molecular pathways related to different cellular origins may provide a biological basis for targeted therapeutic strategies in PCNSL (11). Therefore, predicting pathologic subtyping is likely valuable for characterizing biological heterogeneity within a broader molecular framework. Indeed, early, ideally preoperative, identification of these phenotypes has been suggested to facilitate clinical decision-making (12). Although histopathologic examination after biopsy remains the diagnostic gold standard for PCNSL, surgical resection is not standard practice. Thus, a noninvasive approach that can assist in subtype identification may provide additional clinical benefit.

Magnetic resonance imaging (MRI) is the first-line imaging modality for patients with suspected brain tumors and shows promise for tumor subtype characterization (13, 14). However, studies specifically targeting the preoperative, noninvasive prediction of DEL and GCB in PCNSL remain limited, and conventional imaging assessment alone has limited discriminatory value (4, 15, 16). Radiomics, which enables high-throughput quantitative feature extraction and machine-learning–based integration, has been successfully applied to diagnosis, differential diagnosis, and prognostic evaluation in PCNSL and other brain tumors (17, 18). This study aimed to leverage pretreatment multiparametric MRI radiomics to construct models for prediction of immunohistochemical and molecular phenotypes in PCNSL, including DEL versus non-DEL and GCB versus non-GCB subtypes, with the long-term goal of improving noninvasive risk stratification and clinical management.

## 2 Methods

### 2.1 Study design and patient selection

**Figure 1:**
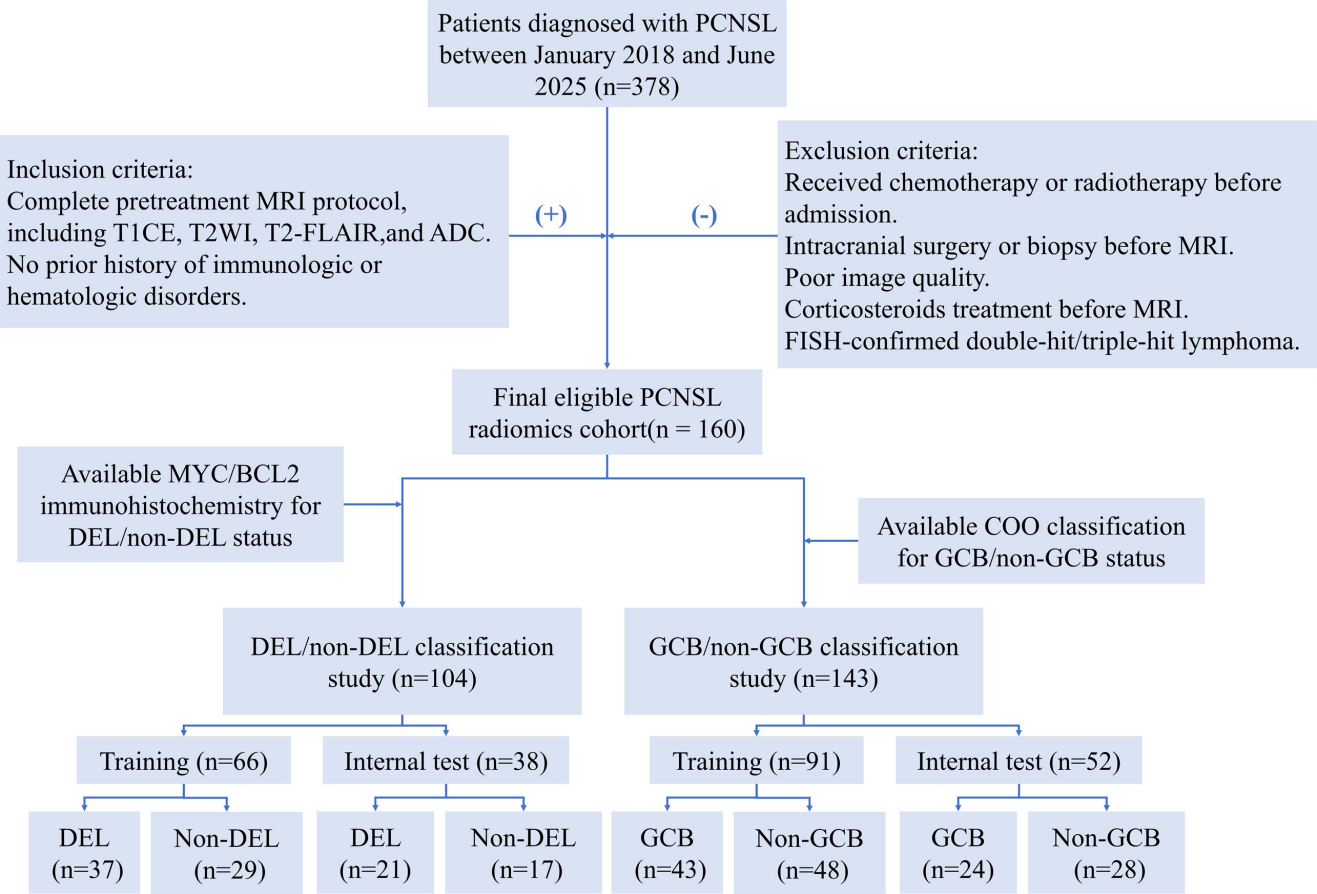
Patient selection flowchart. A total of 160 patients with pathologically confirmed PCNSL were included. Among them, 87 had both DEL and COO information, 17 had only DEL information, and 56 had only COO information, resulting in 104 patients for DEL/non-DEL analysis and 143 patients for GCB/non-GCB analysis.

**Figure 2:**
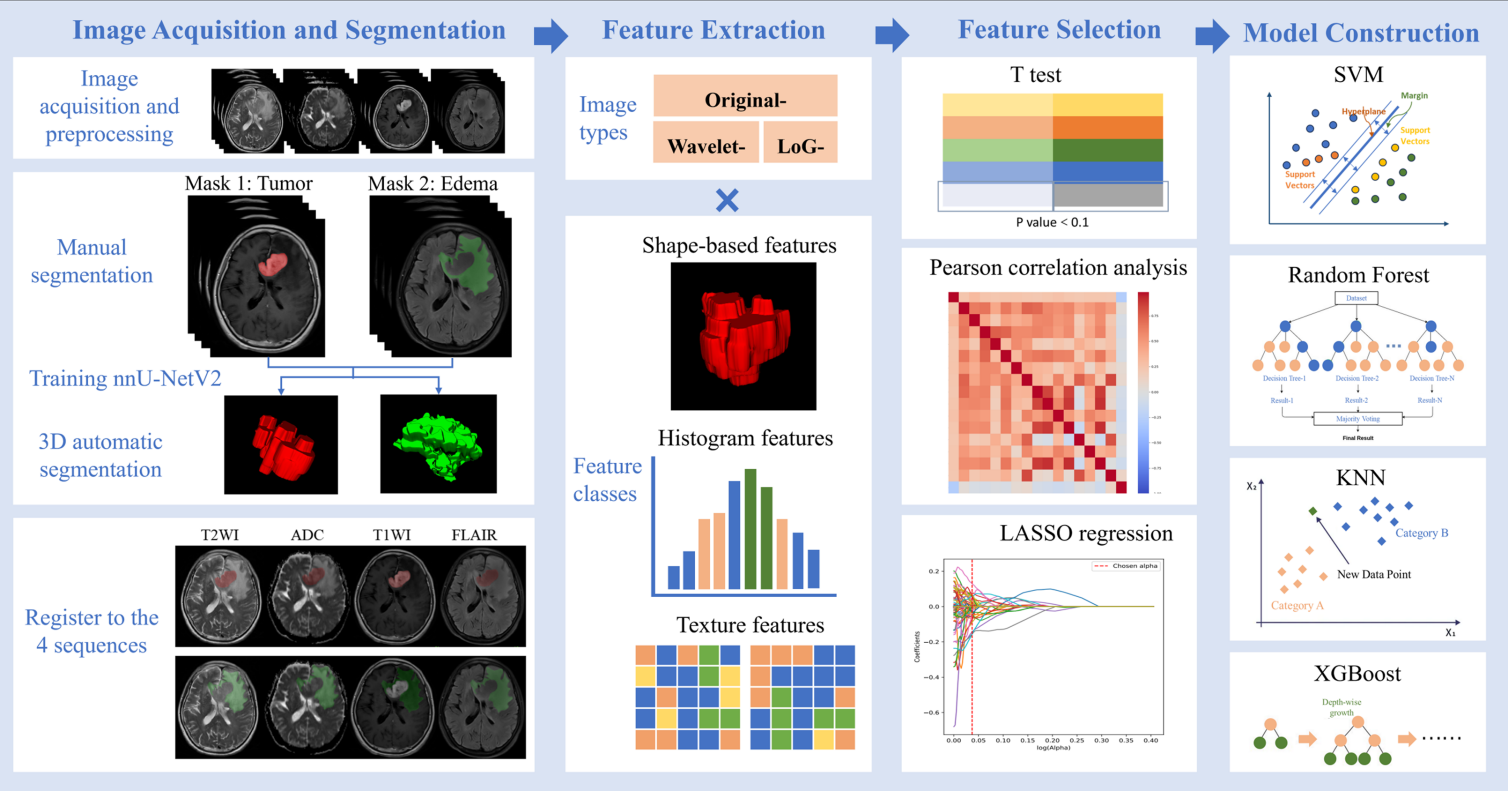
Study design: MRI acquisition and preprocessing, automated segmentation, radiomics feature extraction and selection, and model construction.

This single-center, retrospective study was approved by the Institutional Review Board (IRB) of Beijing Tiantan Hospital, Capital Medical University (Approval No. KY2023-113–02). The requirement for written informed consent was waived by the IRB due to the retrospective nature of the study and the use of anonymized clinical and imaging data. The study was conducted in accordance with the Declaration of Helsinki and its amendments. The study involved 160 out of a total of 378 patients with PCNSL who were admitted to Beijing Tiantan Hospital between January 2018 and June 2025; patient selection flow is summarized in **Figure 1**, and the end-to-end research pipeline is summarized in **Figure 2**.

Inclusion criteria included (1): histopathologic confirmation of PCNSL by stereotactic biopsy or surgery (2); available immunohistochemistry (IHC) showing MYC/BCL2 (double-expressor status) and/or cell-of-origin (GCB vs non-GCB status); and (3) no past history of other hematologic or immune disorders. Exclusion criteria included (1): incomplete clinical data or incomplete MRI protocol (2); poor image quality, including severe artifacts or visually inadequate signal-to-noise ratio, precluding reliable ROI delineation (3); any intervention (biopsy, surgery, radiotherapy, or chemotherapy) performed before MRI; and (4) Corticosteroid treatment before MRI. Image quality was assessed visually during patient screening by two radiologists with more than 5 years of experience in neuroradiologic imaging.

DEL status was defined by IHC as BCL2 expression ≥50% and c-MYC expression ≥40%. Available fluorescent *in situ* hybridization (FISH) results were reviewed, and cases with confirmed double-hit or triple-hit rearrangements were excluded from our research. For the DEL/non-DEL analysis, we included all eligible consecutive patients with available double-expressor status within the study period. Because GCB is relatively uncommon in PCNSL, all eligible GCB cases were included, while non-GCB cases from the same study period were randomly selected using stratified sampling to achieve an approximately balanced GCB/non-GCB cohort for model development. This strategy was used to reduce class imbalance during model training rather than relying on the real-world prevalence of GCB in PCNSL. The reporting of this study adhered to the Checklist for Artificial Intelligence in Medical Imaging (CLAIM) guidelines (19) with the completed checklist presented in **Supplementary Table 1**.

### 2.2 MRI protocol and image preprocessing

MRI was performed on 3.0-T scanners from three manufacturers, all equipped with 8-channel head coils. The scanners included two GE Discovery 750 (GE Healthcare, USA), one Philips Ingenia (Philips Healthcare, the Netherlands), and one Siemens Verio (Siemens Healthineers, Germany). The scanning protocol included T1-weighted (T1WI), T2-weighted (T2WI), T2-Fluid-Attenuated Inversion Recovery (FLAIR), diffusion-weighted imaging (DWI; b=0 and 1000 s/mm²), and contrast-enhanced T1-weighted imaging (T1CE). Apparent diffusion coefficient (ADC) maps were generated on-console as trace ADC from the b0/b1000 DWI using a mono-exponential fit. Detailed acquisition parameters are provided in **Supplementary Table 2**.

To harmonize data across scanners and protocols, all images underwent a standardized preprocessing workflow in Python (3.x): DICOM files for T1CE, T2WI, FLAIR, and ADC were converted to NIfTI using dicom2nifti; low-frequency intensity inhomogeneity was corrected with N4 bias-field correction (SimpleITK); volumes were then resampled to isotropic 1.0×1.0×1.0 mm³ (linear/B-spline interpolation for images; nearest-neighbor for masks). Finally, per-volume z-score normalization was applied ( $z_{\text{i}}=(x_{\text{i}}-\mu )/\sigma$) to produce zero-mean, unit-variance images for downstream radiomics feature extraction.

### 2.3 Demographic and conventional imaging features

The following items were recorded in a standardized case report form: (1) Age and sex; (2) Tumor laterality/location: midline versus hemispheric; (3) Compartment: supratentorial, infratentorial, or both; (4) Multiplicity: solitary versus multifocal; (5) Deep structure involvement: infiltration of periventricular region, basal ganglia, brainstem, or cerebellum (present/absent); (6) Cystic change/necrosis: present/absent; (7) Tumor margin: regular versus irregular; (8) Enhancement pattern on T1CE: homogeneous versus heterogeneous (excluding non-enhancing cystic/necrotic areas); (9) Midline shift: present/absent; (10) Angular sign—a sharply tapering projection extending from the enhancing mass; (11) Dimple (umbilication) sign—focal indentation along the lesion margin; (12) Fist sign—serial, groove-like notches along the tumor edge resembling a clenched fist; (13) Butterfly sign—transcallosal spread producing a symmetric “butterfly” configuration; (14) Purfling sign—a thin, smooth, peripheral enhancing rim (“porcelain-like edging”). Two board-certified radiologists (each with 5 years of CNS imaging experience) independently assessed all MRI studies while blinded to histopathology and outcomes; discrepancies were adjudicated by a senior neuroradiologist with 15 years of experience. All features were defined *a priori* in an imaging manual to ensure consistency across readers.

### 2.4 ROI segmentation

An automatic 3D segmentation model based on nnU-NetV2 (20) was trained on 60 cases selected from the training cohort to avoid data leakage from the test set. Training labels were manually delineated in ITK-SNAP by a trained radiologist. To assess annotation reliability, 20 of the 60 cases were randomly selected and independently re-annotated by a second radiologist in a blinded manner. Details of the training process are provided in the **Supplementary Material**. Two regions were segmented: the enhancing tumor core (excluding macroscopic cystic/necrotic areas) was delineated on T1CE and defined as mask-1, and the peritumoral edema was delineated on FLAIR and defined as mask-2. Model performance on the validation set was quantified using the Dice similarity coefficient (DSC) and the intraclass correlation coefficient (ICC). The training process is illustrated in **Supplementary Figure 1**.

The trained nnU-NetV2 model, including separate models for tumor and edema segmentation, were then applied to the entire dataset for automatic ROI segmentation. The resulting masks were registered to all MRI sequencesusing SimpleITK; a senior neuroradiologist (>15 years experience) reviewed and, when necessary, corrected the segmentation labels. For volumetry, the binary NIfTI masks (tumor/edema) were processed with NiBabel to count non-zero voxels and multiply by voxel size (vx×vy×vz, mm³); volumes were converted to milliliters (mL) to obtain Tumor_Volume and Edema_Volume as conventional imaging features.

### 2.5 Feature extraction and selection

After standardized preprocessing, radiomic features were computed with PyRadiomics version 3.1.0 (IBSI-compliant) from four MRI sequences T1CE, T2WI, FLAIR, and ADC—on mask-1 and mask-2 per patient, yielding eight extractions per case. Discretization used sequence-specific binWidth (T1CE = 20, T2WI = 30, FLAIR = 50, ADC = 50); LoG filters were applied at σ = 1, 2, 3. Radiomic features were extracted from Original, Laplacian of Gaussian (LoG)-filtered, and Wavelet-transformed images. The extracted features were categorized into first-order, shape, and texture features. First-order features describe the distribution of voxel intensity values within the segmented ROI. Shape features quantify the two- and three-dimensional geometric properties of the ROI, including tumor size, contour, and spatial morphology. Texture features characterize the spatial arrangement and heterogeneity of voxel intensities, including features derived from the gray-level co-occurrence matrix (GLCM), gray-level run-length matrix (GLRLM), gray-level size-zone matrix (GLSZM), gray-level dependence matrix (GLDM), and neighboring gray-tone difference matrix (NGTDM). Feature stability was assessed using the ICC, and unstable features with ICC < 0.75 were excluded. To reduce scanner-related batch effects, the extracted radiomic features were harmonized using ComBat, with scanner information used as the batch variable with age and sex included as covariates.

Feature selection was performed only in the training set. The sequence was: (i) variance homogeneity check followed by independent t-tests (retention if *P* < 0.05); (ii) correlation filtering removing highly collinear variables (**|**r| > 0.90; Pearson/Spearman by distribution), with retained features z-score standardized; (iii) least absolute shrinkage and selection operator (LASSO) logistic regression with 10-fold cross validation (CV) to keep non-zero-coefficient features; and (iv) Random Forest (RF) importance with forward, AUC-guided addition (internal 80/20 split) to determine the minimal subset achieving the peak AUC. A detailed rationale for each feature-reduction step and for the sequential multistep strategy is provided in the **Supplementary Methods**.

### 2.6 Radiomics-based model construction

We implemented six candidate classifiers using scikit-learn (https://scikit-learn.org/) and related libraries: support vector machine (SVM), stochastic gradient descent classifier (SGD; logistic loss), k-nearest neighbors (KNN), RF, XGBoost, and LightGBM. Models were trained on selected features using the training cohort, and 5-fold stratified cross-validation was applied to tune hyperparameters. The model with the optimal hyperparameters selected through iterative tuning in the training cohort was evaluated in the independent internal test set using receiver operating characteristic (ROC) curve analysis. Decision curve analysis (DCA) was performed to assess the clinical utility of the predictive model in real-world decision-making. To improve interpretability, Shapley Additive exPlanations (SHAP) analysis was performed to assess the magnitude and direction of each feature’s contribution to the model output.

**Figure 2** summarizes the workflow of radiomics model construction, including MRI acquisition and preprocessing, automated segmentation, radiomics feature extraction and selection, and model construction.

### 2.7 Clinical and radiomics-clinical based model

Clinical and conventional imaging features that differed significantly between the DEL/non-DEL and GCB/non-GCB groups were identified using univariate and multivariable analyses, and were subsequently used to develop clinical prediction models. After constructing the radiomics-only model, additional clinical-only and radiomics-clinical models were built by combining the selected optimal radiomic feature subset and best-performing classifier with the clinical features selected from the training cohort. The performance of three models—the radiomics model, clinical model, and radiomics-clinical model—was then compared to identify the optimal model for PCNSL subtype classification and to evaluate whether clinical variables provided incremental value beyond radiomic features.

### 2.8 Statistical analysis

All analyses were performed in Python 3.9.18. Continuous variables were assessed for normality; non-normally distributed data are presented as median and interquartile range (IQR). The ICC was used to assess inter-observer agreement. In univariate analyses, continuous variables were compared with Student’s t-test and categorical variables with the χ² test. Pearson correlation analysis was used to evaluate redundancy among radiomic features and assist in removing highly collinear variables. Model performance was summarized by ROC-AUC, accuracy (Acc), sensitivity (Sen), specificity (Spe), positive predictive value (PPV), negative predictive value (NPV), and F1-score. Because of the small sample size of the test set, 95% confidence intervals for AUC were estimated using nonparametric bootstrap resampling with 10,000 iterations based on fixed test-set predictions. Given the relatively low prevalence of GCB in real-world PCNSL populations, PPV and NPV were adjusted using Bayes’ theorem assuming a GCB prevalence of 25%. Decision curve analysis was performed to determine whether the radiomics model provided meaningful net clinical benefit. The DeLong test was used to compare the performance of the clinical, radiomics, and radiomics-clinical models. A two-sided *P* < 0.05 was considered statistically significant.

## 3 Results

### 3.1 Baseline characteristics

A total of 160 patients with PCNSL were included. The DEL/non-DEL and GCB/non-GCB analyses were performed in different but overlapping subcohorts based on IHC availability: specifically, 87 patients had both DEL and COO profiles, 17 had DEL status only, and 56 had COO status only. DEL analysis was conducted in 104 patients who had MYC/BCL2 IHC results (58 DEL; 46 non-DEL). Among the 378 initially screened patients, 42 patients had available FISH results, and double-hit or triple-hit lymphoma had been excluded in all these FISH-tested cases. 143 patients had COO status information available (67 GCB; 76 non-GCB), including 128 cases classified by the Hans algorithm.

The DEL/non-DEL dataset was split into a training set (n = 66; DEL = 37, non-DEL= 29) and an independent test set (n = 38; DEL = 21, non-DEL = 17). The COO cohort was split into a training set (n = 91; GCB = 43, non-GCB = 48) and a test set (n = 52; GCB = 24, non-GCB=28).

### 3.2 Training test comparability

**Supplementary Table 3** summarizes baseline characteristics for the DEL vs non-DEL cohorts in the training and test sets. Apart from midline vs hemispheric location, no variable differed significantly between the sets, indicating good comparability. The midline/hemisphere imbalance likely reflects random sampling variability. **Supplementary Table 4** shows baseline characteristics for the GCB vs non-GCB cohorts; distributions of patient and imaging variables were well balanced between the training and test sets with no significant differences (*P* > 0.05).

### 3.3 Comparison between DEL and non-DEL cases

In the DEL vs non-DEL comparison using all available cases, tumor volume was larger in the DEL group (20.1 ± 15.6 mL) than in the non-DEL group (13.4 ± 9.7 mL, *P* = 0.008), while all other variables did not differ significantly (**Table 1**). In the training set (n = 66), univariate logistic regression identified Edema_Volume as significantly associated with DEL (*P* = 0.03); however, when all variables with *P* < 0.10, from univariate analysis, were included in a multivariate analysis, no significant independent predictor remained (**Supplementary Table 5**).

**Table 1:** Comparison of baseline characteristics between DEL and non-DEL subtypes in the PCNSL cohort (n=104).

Variable	DEL (n=58)	Non-DEL (n=46)	*P* value
Age (years)	59.1 ± 11.2	54.8 ± 14.5	**0.09**
Gender			0.57
Male	27 (47%)	24 (52%)	
Female	31 (53%)	22 (48%)	
Tumor Volume (mL)	20.1 ± 15.6	13.4 ± 9.7	0.008
Edema Volume (mL)	57.4 ± 45.2	40.3 ± 45.0	0.06
Location			0.42
Midline	22 (38%)	14 (30%)	
Hemisphere	36 (62%)	32 (70%)	
Tentorial Compartment			0.18
Supratentorial	49 (85%)	33 (72%)	
Infratentorial	3 (5%)	7 (15%)	
Both	6 (10%)	6 (13%)	
Deep Involvement			0.89
Yes	46 (79%)	37 (80%)	
No	12 (21%)	9 (20%)	
Multiple Lesions			0.57
Yes	27 (46%)	24 (52%)	
No	31 (53%)	22 (48%)	
Necrosis			0.20
Yes	30 (52%)	18 (39%)	
No	28 (48%)	28 (61%)	
Tumor Margins			0.62
Irregular	39 (67%)	33 (72%)	
Regular	19 (33%)	13 (28%)	
Enhancement Pattern			0.95
Non-homogeneous	40 (69%)	32 (70%)	
Homogeneous	18 (31%)	14 (30%)	
Angular Sign			0.54
Yes	23 (40%)	21 (46%)	
No	35 (60%)	25 (54%)	
Umbilication Sign			0.08
Yes	28 (48%)	30 (65%)	
No	30 (52%)	16 (35%)	
Fist Sign			0.49
Yes	16 (28%)	10 (22%)	
No	42 (72%)	36 (78%)	
Butterfly Sign			0.15
Yes	9 (16%)	3 (7%)	
No	49 (85%)	43 (94%)	
Purfling Sign			0.62
Yes	11 (19%)	7 (15%)	
No	47 (81%)	39 (85%)	

GCB Germinal Center B-cell-like. Categorical variables are presented as number of patients (percentage). Continuous variables are presented as Mean ± SD. Categorical variables followed by “(Yes)” indicate that the data in that row represent the number and percentage of cases that meet the condition (i.e., are positive). P-values in bold indicate statistical significance (*P* < 0.05).

### 3.4 Comparison between GCB and non-GCB

Comparative analysis of the GCB and non-GCB subtypes revealed several significant differences in baseline characteristics (**Table 2**). Specifically, patients in the GCB subgroup were significantly younger (53.2 ± 16.0 vs. 59.0 ± 11.0 years, P = 0.01) and more likely to be male (75% vs. 42%, *P* < 0.001). In terms of imaging features, GCB tumors demonstrated a higher frequency of infratentorial involvement (16% vs. 5%, *P* = 0.007) and a lower prevalence of the butterfly sign (3% vs. 13%, P = 0.029) compared to the non-GCB subgroup.

**Table 2:** Comparison of baseline characteristics between GCB and non-GCB subtypes in the PCNSL cohort (n=143).

Variable	GCB (n=67)	Non-GCB (n=76)	*P* value
Age (years)	53.2 ± 16.0	59.0 ± 11.0	0.01
Gender			<0.001
Male	50 (74.6%)	32 (42.1%)	
Female	17 (25.4%)	44 (57.9%)	
Tumor Volume (mL)	18.1 ± 28.1	18.3 ± 14.0	0.96
Edema Volume (mL)	50.1 ± 50.9	52.6 ± 43.0	0.75
Location			0.59
Midline	24 (35.8%)	24 (31.6%)	
Hemisphere	43 (64.2%)	52 (68.4%)	
Tentorial Compartment			0.007
Supratentorial	42 (62.7%)	65 (85.5%)	
Infratentorial	11 (16.4%)	4 (5.3%)	
Both	14 (20.9%)	7 (9.2%)	
Deep Involvement			0.66
Yes	54 (80.6%)	59 (77.6%)	
No	13 (19.4%)	17 (22.4%)	
Multiple Lesions			0.69
Yes	34 (50.7%)	36 (47.4%)	
No	33 (49.3%)	40 (52.6%)	
Necrosis			0.11
Yes	28 (41.8%)	42 (55.3%)	
No	39 (58.2%)	34 (44.7%)	
Tumor Margins			0.22
Irregular	44 (65.7%)	57 (75.0%)	
Regular	23 (34.3%)	19 (25.0%)	
Enhancement Pattern			0.72
Non-homogeneous	43 (64.2%)	51 (67.1%)	
Homogeneous	24 (35.8%)	25 (32.9%)	
Angular Sign			0.19
Yes	22 (32.8%)	33 (43.4%)	
No	45 (67.2%)	43 (56.6%)	
Umbilication Sign			0.47
Yes	41 (61.2%)	42 (55.3%)	
No	26 (38.8%)	34 (44.7%)	
Fist Sign			0.23
Yes	21 (31.3%)	17 (22.4%)	
No	46 (68.7%)	59 (77.6%)	
Butterfly Sign			0.029
Yes	2 (3.0%)	10 (13.2%)	
No	65 (97.0%)	66 (86.8%)	
Purfling Sign			0.35
Yes	7 (10.4%)	12 (15.8%)	
No	60 (89.6%)	64 (84.2%)	

PCNSL, Primary Central Nervous System Lymphoma; DEL, Double-Expressor Lymphoma; GBC, Germinal Center B-cell-like. Categorical variables are presented as number of patients (percentage). Continuous variables are presented as Mean ± SD. Categorical variables followed by “(Yes)” indicate that the data in that row represent the number and percentage of cases that meet the condition (i.e., are positive). *P*-values in bold indicate statistical significance (*P* < 0.05).

In the training set (n=91), after univariate screening age and sex were further entered into multivariate regression analysis. Both variables remained significant in the multivariate model (age: *P* = 0.026; sex: *P* = 0.026) and were subsequently included in the clinical model (**Table 3**).

**Table 3:** Univariate and multivariate logistic regression analysis of patient characteristics for predicting GCB subtype (training cohort, n = 91).

Characteristic	Univariate analysis		Multivariate analysis		
	*OR* (95% *CI*)	*P* value	*β* Coefficient	*OR* (95% CI)	*P* value
Age (years)	0.96 (0.93, 0.99)	0.012	-0.04	0.96 (0.92, 0.99)	**0.026**
Gender					
Female	Reference				
Male	2.58 (1.08, 6.19)	0.033	1.08	2.95 (1.14, 7.67)	**0.026**
Tumor Volume (mL)	1.00 (0.99, 1.02)	0.764			
Edema Volume (mL)	1.00 (0.99, 1.01)	0.814			
Location					
Hemisphere	Reference				
Midline	1.21 (0.60, 2.42)	0.592			
Tentorial Compartment					
Supratentorial	Reference				
Infratentorial	4.50 (0.85, 23.91)	0.078	1.22	3.40 (0.59, 19.53)	0.17
Both	3.38 (0.95, 12.03)	0.061	1	2.71 (0.69, 10.67)	0.153
Deep Involvement					
No	Reference				
Yes	1.53 (0.53, 4.38)	0.429			
Multiple Lesions					
No	Reference				
Yes	1.35 (0.59, 3.08)	0.48			
Necrosis					
No	Reference				
Yes	0.52 (0.23, 1.20)	0.128			
Tumor Margins					
Regular	Reference				
Irregular	0.77 (0.30, 1.98)	0.585			
Enhancement Pattern					
Homogeneous	Reference				
Non-homogeneous	1.15 (0.48, 2.80)	0.751			
Angular Sign					
No	Reference				
Yes	0.70 (0.30, 1.62)	0.406			
Umbilication Sign					
No	Reference				
Yes	1.79 (0.76, 4.26)	0.185			
Fist Sign					
No	Reference				
Yes	1.30 (0.53, 3.20)	0.569			
Butterfly Sign					
No	Reference				
Yes	0.29 (0.06, 1.46)	0.132			
Purfling Sign					
No	Reference				
Yes	0.95 (0.29, 3.08)	0.932			

*PCNSL*, Primary Central Nervous System Lymphoma; *GCB*, Germinal Center B-cell-like; *OR*, Odds Ratio; *CI*, Confidence Interval; Ref, Reference category. The multivariate model was constructed by including variables with a *P* < 0.10 from the univariate analysis. Bold P values indicate statistical significance (*P* < 0.05).

### 3.5 PCNSL segmentation model

Training details are provided in the **Supplementary Material**. Regarding the reliability of the reference standard, the blinded 20-case manual re-annotation subset demonstrated exceptional inter-reader agreement, yielding a mean DSC of 0.97 (volume ICC = 0.99) for the enhancing tumor core and 0.91 (volume ICC = 0.98) for the peritumoral edema (**Supplementary Table 6**). An nnU-NetV2–based 3D segmentation network was trained separately for the tumor and edema masks. On the validation set, the model achieved a DSC of 0.90 for the enhancing tumor parenchyma and 0.72 for peritumoral edema (**Supplementary Table 6**).

### 3.6 Feature extraction and selection

For each sequence–ROI combination, 1,130 radiomic features were extracted, yielding a total of 8,956 unique features per patient after merging across four sequences and removing duplicate shape descriptors. To ensure feature reproducibility, a stability analysis was performed on the 60-case training subset; features with an inter-strategy intraclass correlation coefficient (ICC) < 0.75 between manual and automatically assisted segmentations were deemed unstable and excluded. Consequently, 6,806 stable radiomic features were retained for downstream ComBat harmonization and feature selection.

After t-tests, correlation filtering, LASSO regression, and AUC-based calibration, 12 stably selected radiomic features were retained for the DEL versus non-DEL analysis. Using the same feature selection pipeline, seven radiomic features were retained for subsequent COO classification modeling. The names of the selected features are provided in **Table 4**.

**Table 4:** The radiomics feature set retained after screening for modeling the two tasks.

Features for COO analysis	Features for DEL analysis
Edema_T1CE_wavelet-LLL_glszm_SmallAreaLowGrayLevelEmphasis	Edema_T1CE_wavelet-LLH_gldm_LargeDependenceLowGrayLevelEmphasis
Tumor_FLAIR_wavelet-LHH_glrlm_ShortRunHighGrayLevelEmphasis	Tumor_T2_wavelet-LHL_firstorder_Maximum
Tumor_T1CE_wavelet-HLL_glszm_SmallAreaEmphasis	Tumor_T1CE_wavelet-LHH_glszm_SizeZoneNonUniformity
Tumor_ADC_wavelet-HHH_glcm_DifferenceAverage	Edema_ADC_wavelet-LHL_glszm_SmallAreaLowGrayLevelEmphasis
Edema_ADC_log-sigma-2-mm-3D_glszm_SmallAreaLowGrayLevelEmphasis	Tumor_FLAIR_wavelet-HLH_glszm_HighGrayLevelZoneEmphasis
Tumor_ADC_wavelet-LLL_glszm_SizeZoneNonUniformityNormalized	Tumor_FLAIR_wavelet-HLH_glszm_SmallAreaHighGrayLevelEmphasis
Edema_T1CE_wavelet-LLL_glszm_SizeZoneNonUniformityNormalized	Tumor_ADC_wavelet-LLL_glcm_ClusterShade
	Edema_FLAIR_log-sigma-2-mm-3D_firstorder_Range
	Tumor_FLAIR_wavelet-LLL_firstorder_RobustMeanAbsoluteDeviation
	Edema_T1CE_wavelet-LLH_glcm_ClusterTendency
	Edema_T2_log-sigma-2-mm-3D_glrlm_ShortRunHighGrayLevelEmphasis
	Edema_ADC_wavelet-LHL_gldm_DependenceVariance

### 3.7 Radiomics model construction

#### 3.7.1 DEL vs non-DEL

**Figure 3:**
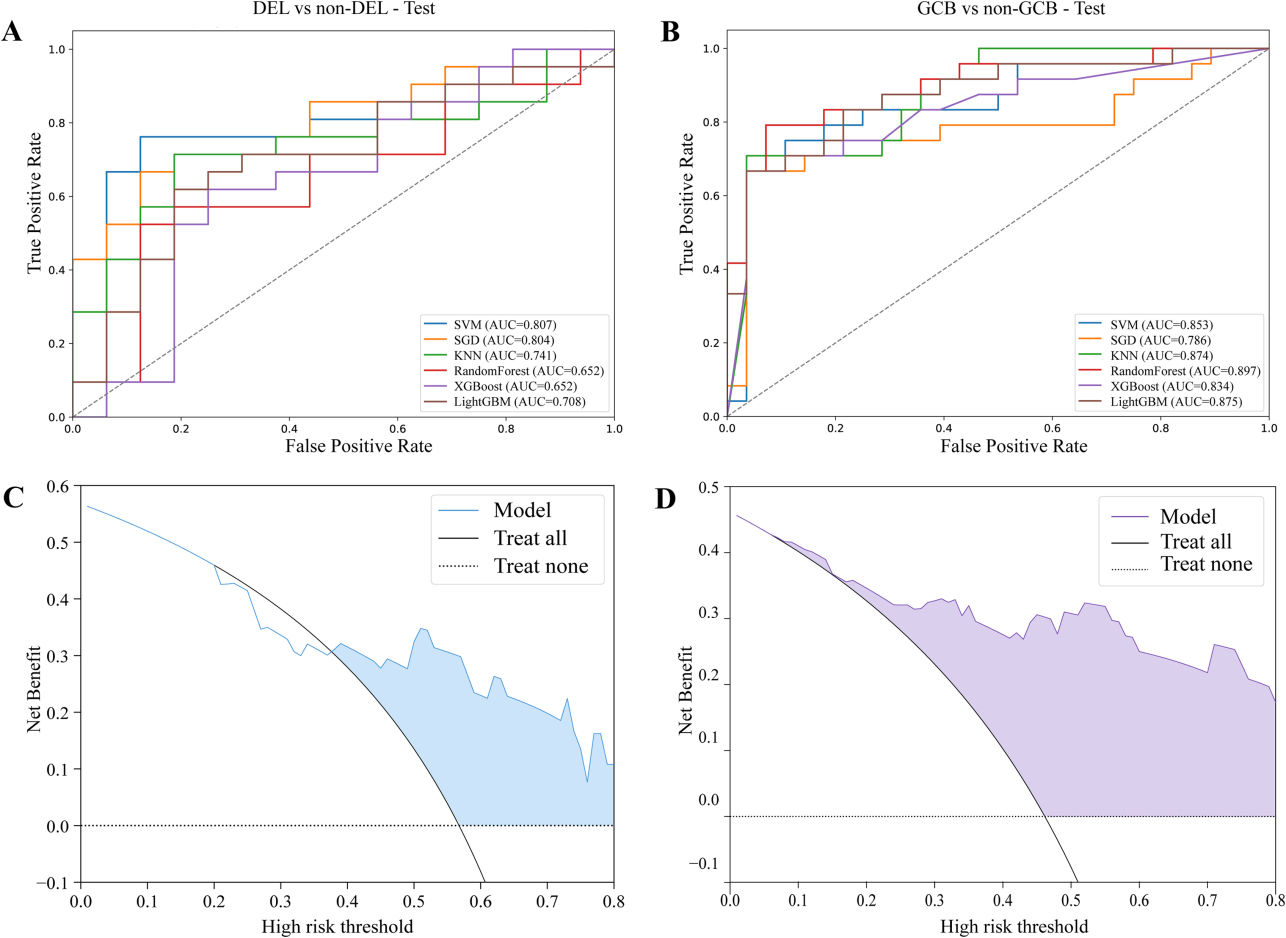
Performance of radiomics models for DEL and COO classification. **(A)** ROC curves of six machine-learning classifiers for DEL versus non-DEL classification in the test set. **(B)** ROC curves of six classifiers for GCB versus non-GCB classification in the test set. **(C)** Decision curve analysis of the DEL radiomics model. **(D)** Decision curve analysis of the GCB/non-GCB radiomics model. DEL, double-expression lymphoma; COO, cell-of-origin; GCB, germinal center B-cell-like; ROC, receiver operating characteristic.

Using the 12 selected features, we trained six classifiers (SVM, SGD, KNN, RF, XGBoost, LightGBM). Model tuning used stratified 5-fold cross-validation in the training cohort. The model performance and the mean 5-fold ROC curve of the training cohort is shown in **Supplementary Table 7** and **Supplementary Figure 2**, and the ROC curve of the internal test set is shown in **Figure 3A**. Among the six classifiers, the SVM classifier achieved the best performance, with an AUC of 0.807 (95% CI, 0.649–0.936). In the test set, this model yielded a sensitivity of 0.714, specificity of 0.750, and overall accuracy of 0.730 (**Table 5**). DCA showed that the radiomics model provided a positive net benefit over the “treat-all” and “treat-none” strategies mainly across intermediate-to-high threshold probabilities.

**Table 5:** Performance of six machine learning models for distinguishing DEL from non-DEL in the held-out internal test set (n=38).

Model	AUC	95% CI	Accuracy	Sensitivity	Specificity	PPV	NPV	F1 score
**SVM**	**0.807**	**0.649–0.936**	**0.730**	**0.714**	**0.750**	**0.779**	**0.680**	**0.745**
SGD	0.804	0.652–0.930	0.703	0.762	0.625	0.715	0.680	0.738
KNN	0.741	0.565–0.893	0.730	0.762	0.687	0.750	0.700	0.756
RF	0.652	0.462–0.829	0.622	0.714	0.500	0.638	0.586	0.674
XGBoost	0.652	0.452–0.833	0.622	0.619	0.625	0.671	0.570	0.644
LightGBM	0.708	0.527–0.869	0.703	0.714	0.687	0.738	0.660	0.726

DEL, Double-Expressor Lymphoma; SVM, Support Vector Machine; SGD, Stochastic Gradient Descent (L2-regularized logistic classifier); KNN, K-Nearest Neighbors; RF, Random Forest; XGBoost, extreme Gradient Boosting; GBM, Gradient Boosting Machine; 95% CI: 95% confidence interval estimated by bootstrap resampling. PPV, positive predictive value; NPV, negative predictive value; F1 score, harmonic mean of precision and sensitivity.

Bold values indicate the performance metrics of the best-performing model.

#### 3.7.2 GCB/non-GCB

The ROC curves of the six machine-learning models built with seven radiomic features in the training and test cohorts are shown in **Supplementary Figure 2** and **Figure 3B**, respectively. The RF achieved the highest test AUC 0.897 (95% CI 0.796–0.973), with sensitivity of 0.792, specificity of 0.893, and accuracy of 0.846. Because the real-world prevalence of GCB is approximately 25%, which is lower than that in our study cohort (47%), PPV and NPV were adjusted using Bayes’ theorem. After prevalence adjustment, the PPV and NPV of the RF classifier were approximately 0.711 and 0.928, respectively. **Table 6** summarizes the performance of the COO classifiers, including both unadjusted and prevalence-adjusted PPV and NPV. **Supplementary Table 8** shows the performance in the training set. DCA demonstrated consistently higher net benefit than the “treat-all” and “treat-none” strategies across a broad range of threshold probabilities, indicating favorable clinical utility for individualized risk stratification.

**Table 6:** Performance of six machine learning models for distinguishing GCB from non-GCB subtype in the held-out internal test set (n=52).

Model	AUC	95% CI	Acc	Sen	Spe	PPV	NPV	P-PPV	P-NPV	F1 score
SVM	0.853	0.729–0.953	0.808	0.750	0.857	0.818	0.800	0.636	0.911	0.783
SGD	0.786	0.635–0.916	0.788	0.708	0.857	0.810	0.774	0.623	0.898	0.756
KNN	0.874	0.764–0.956	0.808	0.708	0.893	0.850	0.781	0.688	0.902	0.773
**RF**	**0.897**	**0.796–0.973**	**0.846**	**0.792**	**0.893**	**0.864**	**0.833**	**0.711**	**0.928**	**0.826**
XGBoost	0.834	0.709–0.940	0.750	0.750	0.750	0.720	0.778	0.500	0.900	0.735
LightGBM	0.875	0.765–0.960	0.769	0.750	0.786	0.750	0.786	0.538	0.904	0.750

GCB, Germinal Center B-cell-like; SVM, Support Vector Machine; SGD, Stochastic Gradient Descent (L2-regularized logistic classifier); KNN, K-Nearest Neighbors; RF, Random Forest; XGBoost, extreme Gradient Boosting; GBM, Gradient Boosting Machine; Acc, Accuracy; Sen, Sensitivity; Spe, Specificity. PPV, positive predictive value; NPV, negative predictive value; F1 score, harmonic mean of precision and sensitivity. P-PPV and P-NPV, prevalence-adjusted PPV and NPV were calculated using Bayes’ theorem based on the observed GCB prevalence of approximately 25% in our center. GCB was considered the positive class.

Bold values indicate the performance metrics of the best-performing model.

### 3.8 Feature interpretability analysis

**Figure 4:**
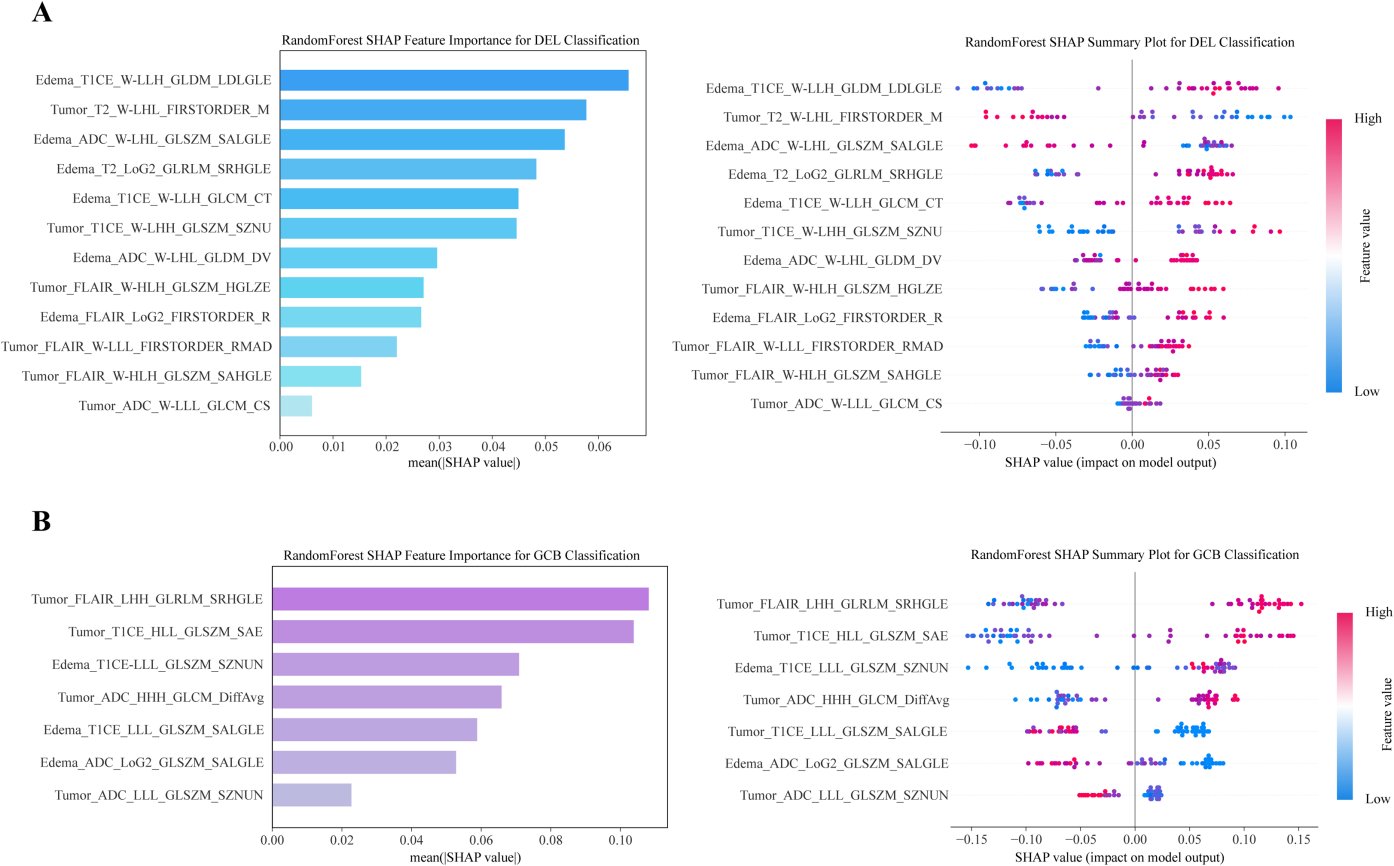
SHAP-based interpretation of radiomics models. **(A)** SHAP feature-importance plot and summary plot for DEL classification. **(B)** SHAP feature-importance plot and summary plot for GCB/non-GCB classification. SHAP values indicate the magnitude and direction of each feature’s contribution to model prediction. SHAP, Shapley additive explanations.

To provide a consistent *post-hoc* interpretation of the selected radiomics features, SHAP analysis was performed using a RF classifier trained with the same feature panel and data split (**Figure 4**). In the GCB classification task, SHAP analysis identified Tumor-FLAIR-LHH-GLRLM-SRHGLE and Tumor-T1CE-HLL-GLSZM-SAE as the two most influential features. Higher values of both features shifted the model output toward GCB classification, indicating positive contributions to GCB prediction. In the DEL classification task, the two most influential SHAP features were Edema-T1CE-W-LLH-GLDM-LDLGLE and Tumor-T2-W-LHL-FirstOrder-Maximum. Higher values of the edema-derived T1CE GLDM feature contributed positively to DEL prediction, whereas higher values of the tumor-core T2 first-order maximum feature contributed negatively to DEL prediction. For the DEL task, this SHAP analysis was used as a *post-hoc* feature-level interpretability analysis and was not intended to replace the final SVM classifier.

### 3.9 Radiomics-clinical model construction

For the DEL/non-DEL task, no independent clinical or conventional imaging predictor remained significant in the training cohort; therefore, no radiomics-clinical model was constructed. For the GCB/non-GCB task, age and sex were retained as independent clinical predictors and used to build the clinical model.

**Figure 5:**
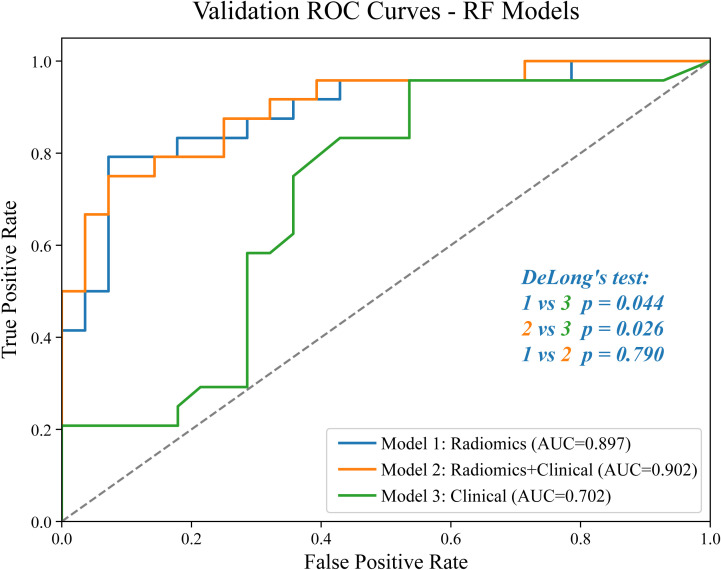
Comparison of test-set ROC curves among the radiomics, clinical-radiomics, and clinical models for GCB/non-GCB classification, with model performance compared using DeLong tests.

Using the RF classifier, the clinical, radiomics, and combined clinical-radiomics models achieved test AUCs of 0.702, 0.897, and 0.902, respectively. Both the radiomics and combined models significantly outperformed the clinical model on DeLong testing (*P* = 0.044 and *P* = 0.026, respectively). Although the combined model yielded the highest AUC, its improvement over the radiomics model alone was not statistically significant (*P* = 0.790) (**Figure 5**).

## 4 Discussion

We have presented our results of a multiparametric MRI-based radiomics study on 160 pathologically confirmed PCNSL patients retrospectively analyzing DEL and non-DEL, GCB and non-GCB cases.

It is important to clarify the distinction between double-expressor lymphoma and double-/triple-hit lymphoma. DEL is defined by immunohistochemical co-expression of MYC and BCL2 proteins, whereas double-/triple-hit lymphoma is genetically defined by MYC rearrangement with concurrent BCL2 and/or BCL6 rearrangements. These two concepts have sometimes been used inconsistently in the literature (21), but they should not be considered interchangeable (22–24). In the 5th edition of the WHO classification, high-grade B-cell lymphoma with MYC and BCL2 rearrangements is recognized as a distinct genetically defined entity, reflecting a biologically more specific molecular subtype (25, 26). Ideally, such cases should be identified by FISH or other molecular assays and analyzed separately from IHC-defined DEL. However, in the present retrospective cohort, FISH results were available only for a subset of patients; therefore, we could not completely exclude rare underlying double-hit or triple-hit cases from the DEL group. This may have introduced some biological heterogeneity into the target class (16). Future studies with systematic molecular testing are warranted to further investigate the overlap between DEL and double-/triple-hit PCNSL and to determine whether these entities differ in imaging characteristics and clinical outcomes.

In this study, COO classification was mainly based on the Hans IHC algorithm using CD10, BCL6, and MUM1/IRF4, which is widely used in routine pathological practice as a practical surrogate for gene expression profiling (6). However, this approach does not fully replace gene expression profiling (GEP)-based molecular classification (5, 27), because it may introduce misclassification bias and generally merges ABC and unclassified cases into the non-GCB group. Therefore, our COO model should be interpreted as predicting the routinely defined GCB/non-GCB immunophenotype rather than the strict GEP-defined molecular subtype. In PCNSL, GCB cases are generally uncommon, accounting for approximately 15%–30% in previous reviews (15, 28, 29); and approximately 25%. in our institutional cohort. We used stratified sampling to mitigate class imbalance during model development, therefore, the observed predictive values may not directly reflect those in routine clinical practice. In fact, PPV and NPV were recalibrated according to the institutional GCB prevalence of 25% using Bayes’ theorem. For example, the prevalence-adjusted PPV was calculated as:


$$PPV_{\text{adjusted}}=\frac{Sensitivity\times Prevalence}{Sensitivity\times Prevalence+(1-Specificity)\times (1-Prevalence)}$$

so that the reported predictive values better reflected the expected performance of the model in the target clinical population.

The prognostic and treatment-predictive significance of pathologic subtypes in PCNSL remains controversial. Although several studies have suggested that patients with GCB-type PCNSL may have more favorable survival outcomes (8, 9), other cohorts, including those reported by Kreher et al. (28) and Radotra et al. (30) found no significant association between the GCB/non-GCB phenotype and overall survival. In contrast, the association between DEL status and adverse prognosis in PCNSL has been more consistently recognized (3, 4, 31, 32). Regarding treatment response, most evidence on the relationship between DLBCL pathologic subtypes and therapeutic sensitivity derives from systemic DLBCL (33). However, systemic DLBCL and PCNSL are treated with different standard strategies (R-CHOP-based immunochemotherapy vs high-dose methotrexate-based regimens); therefore, findings from systemic DLBCL cannot be directly extrapolated to PCNSL. Nevertheless, emerging studies suggest that genetic and pathologic PCNSL phenotypes may reflect distinct biologic pathways and potential therapeutic vulnerabilities (12, 34). Thus, identifying DEL and GCB/non-GCB subtypes remains clinically valuable for biologic characterization, risk stratification, and future individualized treatment strategies.

Several groups have previously explored radiomics/AI to anticipate PCNSL phenotypes before biopsy. Liu et al. (35) trained multiparametric MRI radiomics models to detect DEL in a 40-patient single-center cohort; their best combinations (SVM-linear and logistic regression using four sequences) reported an AUC of 0.92. Similarly, Liu et al. (15) investigated multiparametric MRI radiomics for COO subtype classification in 186 patients with PCNSL. Radiomic features were extracted from a single ROI encompassing both the tumor and peritumoral edema. Using logistic regression and linear-kernel support vector machine models, the ADC+CE-T1WI radiomics model achieved the best performance for COO subtype classification, with an AUC of 0.867 in the independent validation cohort. Moreover, radiomics has been successfully applied to other IHC phenotypes in PCNSL; for example, Zhao et al. developed an interpretable radiomics-based random forest model to predict Ki-67 expression status in PCNSL, achieving a test AUC of 0.84, suggesting that quantitative MRI features can capture biologically meaningful heterogeneity in this disease.

Currently, there are no widely accepted conventional imaging biomarkers for pathologic subtypes of PCNSL. In our full-cohort baseline comparison, DEL tumors were significantly larger than non-DEL tumors (mean volume 20.1 vs 13.4 mL), and in the training set edema volume was significantly associated with DEL on univariate analysis—findings consistent with a more aggressive/infiltrative phenotype, as previously described (4, 36). However, no significant differences were observed between DEL and non-DEL tumors in other conventional MRI features, including tumor margin, necrosis or cystic change, mass effect, angular sign, fist sign, or butterfly sign, which is also broadly consistent with previous observations (14, 35, 37). For COO classification, Zeng et al. (29) investigated conventional MRI differences between GCB and non-GCB subtypes in 53 GCB and 107 non-GCB PCNSL cases. They reported that non-GCB tumors were significantly more likely to show irregular margins, solitary lesions, cystic or necrotic changes, the fist sign, and more severe peritumoral edema. Our findings were not entirely consistent with these observations. In our cohort, non-GCB tumors showed a numerical tendency toward solitary presentation (52.6% vs. 49.3%), cystic or necrotic change (55.3% vs. 41.8%), irregular margins (75.0% vs. 65.7%), and greater edema volume (52.6 ± 43.0 vs. 50.1 ± 50.9 mL), but none of these differences reached statistical significance. In addition, the fist sign was more frequently observed in the GCB group in our cohort (31.3% vs. 22.4%). The significant conventional imaging findings in our study included a higher frequency of the butterfly sign in non-GCB tumors and more frequent infratentorial involvement in GCB tumors. However, infratentorial involvement did not remain significant after multivariable adjustment and was therefore not included in the clinical model. Regarding demographic characteristics, GCB cases were significantly younger than non-GCB cases (53.2 ± 16.0 vs 59.0 ± 11.0 years) and were more frequently male (74.6% vs 42.1%), a trend that is in line with previous studies (15, 29).

We used SHAP analysis to interpret feature importance and directionality (38). For DEL classification, four of the top five radiomic features were derived from the peritumoral edema region, highlighting the importance of peritumoral information in identifying the MYC/BCL2 double-expressor phenotype. This finding is consistent with our baseline imaging analysis and suggests that edema-related features may provide complementary information for DEL classification (39). The top-ranked feature was Edema_T1CE_W-LLH_GLDM_LDLGLE, for which higher values contributed positively to DEL prediction. LDLGLE generally reflects the distribution of large dependent low-gray-level regions (40, 41); when derived from contrast-enhanced T1WI of the edema region, this feature may capture heterogeneous low-enhancement or non-enhancing patterns in the peritumoral area. These patterns may be related to DEL-associated microenvironmental alterations, such as more infiltrative edema, blood–brain barrier disruption, or heterogeneous enhancement (42, 43). The second-ranked feature was Tumor_T2_W-LHL_FIRSTORDER_Mean, for which lower values contributed to DEL prediction. This feature reflects the mean intensity of the wavelet-transformed T2WI signal within the tumor core, suggesting that DEL tumors may have distinct intratumoral T2 signal distributions, potentially associated with higher cellularity, reduced free-water content, or more complex tissue architecture (42, 44). For COO classification, the top two features were both derived from the tumor core, and higher values of both features contributed to GCB prediction. The most important feature, Tumor_FLAIR_LHH_GLRLM_SRHGLE, emphasizes short-run high-gray-level patterns, corresponding to dense, localized high-signal texture variations on FLAIR (40, 41). This suggests that GCB-related imaging patterns may be associated with focal intratumoral FLAIR hyperintensity heterogeneity. The second-ranked feature, Tumor_T1CE_HLL_GLSZM_SAE, is a GLSZM feature that emphasizes small homogeneous gray-level zones. When derived from contrast-enhanced T1WI, this feature may reflect fine-scale enhancement heterogeneity, such as small enhancing patches or localized variations in enhancement within the tumor core. Together, these findings suggest that DEL-related signatures are strongly influenced by the peritumoral microenvironment, whereas COO-related signatures may be more closely associated with intratumoral texture and enhancement heterogeneity.

Algorithm choice also merits further comment. SVM demonstrated superior diagnostic performance for DEL prediction, likely related to its multi-dimensional hyper-plane efficiency in linear-margin separation (45). For the GCB task, the tree-ensemble architecture of RF successfully captured the non-linear interactions among multicontrast feature vectors, minimizing over-fitting under a low events-per-variable (EPV) condition (46). However, the performance differences among candidate machine-learning algorithms were generally modest; our aim was not to demonstrate the intrinsic superiority of a specific classifier, but rather to identify the best-performing model under the finalized feature-processing pipeline.

Several design features strengthen the credibility and potential clinical utility of our findings. First, we employed an automated 3D segmentation pipeline using nnU-NetV2 (20), which can improve segmentation efficiency and reduce inter-reader variability, thereby facilitating future large-scale PCNSL studies. Second, the pipeline integrated multiparametric MRI (T1CE/T2/FLAIR/ADC) (42) and analyzed both tumor and peritumoral edema, leveraging microenvironmental signals often missed by ROI-core-only approaches. Third, we compared six machine-learning classifiers, including both linear and nonlinear algorithms, enabled selection of the optimal classifier for each task and reduced dependence on a single algorithm.

Study limitations require acknowledgement. First, this was a single-center, retrospective study; prospective multicenter validation is needed to test generalizability across diverse patients and protocols. Second, the automatic segmentation performance for peritumoral edema was moderate, likely due to its typically thin, irregular, and ill-defined boundaries in PCNSL. Although all automatically generated masks were reviewed and corrected by a senior radiologist before radiomic feature extraction, edema-derived radiomic features may still be affected by segmentation uncertainty. Another limitation is that occult double-hit or triple-hit lymphomas could not be completely excluded in patients without FISH data. Although rare in PCNSL, such cases may introduce target-class heterogeneity into the DEL model and should be addressed in future studies with systematic molecular testing (2, 3).

In summary, multiparametric MRI-based radiomics integrating enhancing tumor and peritumoral features enabled preoperative discrimination of DEL and COO subtypes of PCNSL. An SVM-based model performed best for DEL and a RF model best for COO; clinical variables added limited incremental value beyond radiomics. These findings suggest that MRI-derived radiomic features may reflect biologic heterogeneity in PCNSL and may support preoperative risk stratification and individualized treatment planning, although larger multicenter prospective studies are needed for validation.

## Data Availability

The raw data supporting the conclusions of this article will be made available by the authors, without undue reservation.
