# Supplementary material for: Preoperative phenotypic stratification of primary central nervous system lymphoma using multiparametric MRI-based radiomics: prediction of germinal center B-cell-like and double-expression status
Source: Front Oncol. 2026 Jun 29;16:1822040. doi: 10.3389/fonc.2026.1822040 (PMC13357172; doi:10.3389/fonc.2026.1822040)
Supplement: Supplementary file 1 [file DataSheet1.doc]

**Training of nnU-Net–Based Automated Segmentation Models for Enhancing Tumor and Peritumoral Edema**

Two radiologists with 5 years of experience in neuroradiology and imaging research manually segmented and reviewed the PCNSL lesions using ITK-SNAP software. Reader 1 manually segmented 60 randomly selected cases, which were subsequently used to train the automated segmentation models. These cases were excluded from the test set of the downstream classification models to avoid data leakage. To assess the reliability of manual annotations, 20 cases were randomly selected from these 60 cases and independently re-annotated by Reader 2 in a blinded manner. The Dice similarity coefficient (DSC) and intraclass correlation coefficient (ICC) were calculated to evaluate inter-reader agreement and to support the use of Reader 1 annotations as the ground truth for model training. As shown in Table S6, inter-reader agreement was high, with DSC and volume ICC values of 0.97 and 0.99 for enhancing tumor segmentation, and 0.91 and 0.98 for peritumoral edema segmentation, respectively.

Automated segmentation of enhancing tumor and peritumoral edema was performed using two separately trained nnU-Net models. For each model, segmentation was formulated as a binary voxel-level classification task, with background labeled as 0 and the target region labeled as 1. Sixty manually annotated MRI cases were used for training. Single-channel MRI volumes in NIfTI format were processed using SimpleITKIO. The 3D full-resolution nnU-Net configuration was used, with Z-score normalization, a patch size of 16 × 320 × 320 voxels, and a batch size of 2. The network was a PlainConvUNet with seven resolution stages, instance normalization, and LeakyReLU activation.

Both models were trained for 50 epochs with an initial learning rate of 0.01 and progressive decay to 0.0003. For the enhancing tumor model, the training and validation losses decreased to −0.84 and −0.86, respectively, and the pseudo Dice increased to 0.91–0.93, with a final moving-average pseudo Dice of 0.90. For the peritumoral edema model, the final training and validation losses were −0.624 and −0.668, respectively; the pseudo Dice increased from 0.512 to 0.791, with a final moving-average pseudo Dice of 0.746 (Figure S1).

Compared with manual segmentation by Reader 1, the automated segmentation models achieved a DSC of 0.90 ± 0.13 for enhancing tumor and 0.72 ± 0.13 for peritumoral edema. The corresponding volume ICCs were 0.99 and 0.97, respectively (Table S2), indicating excellent agreement in volumetric measurements despite lower spatial overlap for peritumoral edema segmentation.

**Rationale for the multistep feature-reduction strategy**

Radiomics analysis typically generates a high-dimensional feature space, especially when multiple MRI sequences, image filters, and regions of interest are analyzed simultaneously. To reduce the risk of overfitting and improve model stability, we used a sequential multistep feature-reduction strategy, with each step designed to address a specific methodological issue.

First, univariate statistical testing was used as an initial coarse screening step to remove features with minimal marginal association with the target label. This step reduced the dimensionality of the feature space before multivariable modeling. Second, correlation filtering was applied to eliminate highly collinear features. When two features were strongly correlated, retaining both would provide limited additional information but could increase model instability and redundancy. Third, LASSO logistic regression with cross-validation was used as a supervised shrinkage method to identify features with non-zero predictive contribution while penalizing unnecessary variables. Finally, Random Forest feature importance with forward AUC-guided addition was used to evaluate the incremental predictive value of candidate features and to determine a compact feature subset that achieved the best interna performance.

These steps were combined because they address complementary aspects of high-dimensional feature selection: marginal relevance, redundancy, supervised sparsity, and incremental predictive contribution. All feature-selection procedures were performed exclusively within the training cohort, and the locked feature subset was subsequently applied to the independent test cohort to minimize information leakage and reduce optimism in model evaluation.

**Table S1. CLAIM checklist.**

| **Section** | **No.** | **CLAIM item** | **Page / Line** | **Status** |
| --- | --- | --- | --- | --- |
| Title/Abstract | 1 | AI methodology identification | Page 1, lines 1–43 | Yes |
| Abstract | 2 | Structured abstract | Pages 1–2, lines 12–43 | Yes |
| Introduction | 3 | Scientific/clinical background | Pages 2–4, lines 48–72 | Yes |
| Introduction | 4 | Study aims/objectives | Page 4, lines 72–76 | Yes |
| Methods: Study design | 5 | Retrospective/prospective design | Page 4, lines 77–86 | Yes |
| Methods: Study design | 6 | Study goal | Pages 4–9, lines 72–76 and 195–217 | Yes |
| Methods: Data | 7 | Data source | Page 4, lines 81–86 | Yes |
| Methods: Data | 8 | Inclusion/exclusion criteria | Pages 4–5, lines 87–107 | Yes |
| Methods: Data | 9 | Data preprocessing | Pages 5–6, lines 122–129 | Yes |
| Methods: Data | 10 | Selection of data subsets | Pages 6–8, lines 130–194 | Yes |
| Methods: Data | 11 | De-identification methods | Page 4, lines 77–81 | Yes |
| Methods: Data | 12 | Handling of missing data | Page 5, lines 93–95 | Yes |
| Methods: Data | 13 | Image acquisition protocol | Pages 5–6, lines 111–120; Supplementary Table S2 | Yes |
| Methods: Reference standard | 14 | Reference standard definition | Pages 4–7, lines 87–169 | Yes |
| Methods: Reference standard | 15 | Rationale for reference standard | Pages 16–17, lines 360–384 | Yes |
| Methods: Reference standard | 16 | Source of annotations | Page 7, lines 146–169 | Yes |
| Methods: Reference standard | 17 | Annotation of test set | Page 7, lines 160–169 | Yes |
| Methods: Reference standard | 18 | Inter-/intra-reader variability | Pages 6–7 and 12–13, lines 140–169 and 283–292 | Yes |
| Methods: Data partitions | 19 | Partition assignment | Page 11, lines 240–256 | Yes |
| Methods: Data partitions | 20 | Disjoint partitions | Page 7, lines 146–149 | Yes |
| Methods: Testing data | 21 | Intended sample size | Page 11, lines 240–256 | Yes |
| Methods: Model | 22 | Detailed model description | Pages 7–10, lines 146–217 | Yes |
| Methods: Model | 23 | Software/frameworks/packages | Pages 5–10, lines 122–238 | Yes |
| Methods: Model | 24 | Initialization of parameters | Not explicitly reported | No |
| Methods: Training | 25 | Training approach | Pages 7–10, lines 146–217 | Yes |
| Methods: Training | 26 | Selection of final model | Pages 9–10 and 13–15 | Yes |
| Methods: Training | 27 | Ensembling techniques | Not applicable | NA |
| Methods: Evaluation | 28 | Performance metrics | Page 10, lines 223–238 | Yes |
| Methods: Evaluation | 29 | Statistical significance/uncertainty | Page 10, lines 223–238 | Yes |
| Methods: Evaluation | 30 | Robustness/sensitivity analysis | Pages 8–9, lines 183–194 | Yes |
| Methods: Evaluation | 31 | Explainability methods | Pages 9 and 14–15, lines 203–206 and 333–345 | Yes |
| Methods: Evaluation | 32 | Evaluation on internal data | Pages 13–15, lines 311–359 | Yes |
| Methods: Evaluation | 33 | External testing | Pages 16–17, lines 360–384 | No |
| Methods: Evaluation | 34 | Clinical trial registration | Not applicable | NA |
| Results: Data | 35 | Included/excluded cases | Page 11, lines 240–256; Figure 1 | Yes |
| Results: Data | 36 | Demographics by partition | Pages 11–12, lines 257–282 | Yes |
| Results: Model performance | 37 | Performance metrics and uncertainty | Pages 13–15, lines 311–359 | Yes |
| Results: Model performance | 38 | Diagnostic precision estimates | Pages 13–15, lines 311–359 | Yes |
| Results: Model performance | 39 | Failure analysis | Not explicitly reported | No |
| Discussion | 40 | Study limitations | Pages 16–18, lines 360–434 | Yes |
| Discussion | 41 | Implications for practice | Pages 17–18, lines 397–434 | Yes |
| Other information | 42 | Protocol/technical details availability | Supplementary Material | Yes |
| Other information | 43 | Availability of software/model/data | Declarations | Yes |
| Other information | 44 | Funding/support | Declarations | Yes |

**Table S2. Detailed MRI scan sequence parameters.**

| **CE-T1WI acquisition parameters for each MRI scanner** | | | | | | |
| --- | --- | --- | --- | --- | --- | --- |
| MRI scanner | TR (ms) | TE (ms) | for | Slice spacing (mm) | FOV | Matrix |
| GE Discovery MR 750 | 2,804 | 19 | 6.50 | 1.00 | 240 | 512 × 512 |
| Siemens Verio | 1,900 | 8.6 | 6.50 | 1.50 | 240 | 512 × 496 |
| Philips Ingenia | 2,000 | 20 | 6.00 | 1.00 | 230 | 512 × 512 |
| **DWI acquisition parameters for each MRI scanner** | | | | | | |
| MRI scanner | TR (ms) | TE (ms) | Slice thickness (mm) | Slice spacing (mm) | FOV | Matrix |
| GE Discovery MR 750 | 2300 | 63.20 | 6.50 | 1.00 | 240 | 256 × 256 |
| Siemens Verio | 4100 | 98.00 | 6.50 | 1.50 | 240 | 156× 156 |
| Philips Ingenia | 2338.51 | 65.97 | 6.00 | 1.00 | 228 | 144× 144 |
| **T2WI acquisition parameters for each MRI scanner** | | | | | | |
| MRI scanner | TR (ms) | TE (ms) | Slice thickness (mm) | Slice spacing (mm) | FOV | Matrix |
| GE Discovery MR 750 | 7385.03 | 106.49 | 6.50 | 1.00 | 240 | 512 × 512 |
| Siemens Verio | 4500 | 99 | 6.50 | 1.50 | 240 | 512 × 496 |
| Philips Ingenia | 3100 | 114.21 | 6.00 | 1.00 | 230 | 512 × 512 |

**Table S3.** Training and test comparability of baseline characteristics for the DEL vs non-DEL.

| **Clinical information**  **and MRI features** | **Total DEL+ non-DEL (n = 104)** | **Training cohort (n = 66)** | **Test cohort**  **(n = 38)** | ***P* value** |
| --- | --- | --- | --- | --- |
| **Age (years)** | 57.2 ± 12.9 | 57.2 ± 12.4 | 57.2 ± 14.0 | 0.76 |
| **Gender** |  |  |  | 0.94 |
| Male | 51 (49%) | 30 (45.5%) | 21 (55%) |  |
| Female | 53 (51%) | 36 (54.5%) | 17 (45%) |  |
| **Tumor Volume (mL)** | 17.1 ± 13.7 | 17.5 ± 140 | 16.5 ± 13.4 | 0.63 |
| **Edema Volume (mL)** | 49.8 ± 45.7 | 47.1 ± 41.0 | 54.6 ± 53.0 | 0.89 |
| **Location** |  |  |  | 0.012 |
| Midline | 36 (35%) | 17 (26%) | 19 (50%) |  |
| Hemisphere | 68 (65%) | 49 (74%) | 19 (50%) |  |
| **Tentoria**  **Compartment** |  |  |  | 0.85 |
| Supratentorial | 82 (79%) | 52 (79%) | 30 (79%) |  |
| Infratentorial | 10 (10%) | 7 (11%) | 3 (8%) |  |
| Both | 12 (11%) | 7 (10%) | 5 (13%) |  |
| **Deep Involvement** |  |  |  | 0.50 |
| Yes | 83 (80%) | 54 (82%) | 29 (76%) |  |
| No | 21 (20%) | 12 (18%) | 9 (24%) |  |
| **Multiple Lesions** |  |  |  | 0.80 |
| Yes | 51 (49%) | 33 (50%) | 18 (47%) |  |
| No | 53 (51%) | 33 (50%) | 20 (53%) |  |
| **Necrosis** |  |  |  | 0.85 |
| Yes | 48 (46%) | 30 (45.5%) | 18 (47%) |  |
| No | 56 (54%) | 36 (54.5%) | 20 (53%) |  |
| **Midline Shift** |  |  |  | 0.89 |
| Yes | 42 (40%) | 27 (41%) | 15 (39.5%) |  |
| No | 62 (60%) | 39 (59%) | 23 (60.5%) |  |
| **Angular Sign** |  |  |  | 0.66 |
| Yes | 44 (42%) | 29 (44%) | 15 (39.5%) |  |
| No | 60 (58%) | 37 (56%) | 23 (60.5%) |  |
| **Umbilication Sign** |  |  |  | 0.43 |
| Yes | 58 (56%) | 40 (61%) | 18 (47%) |  |
| No | 46 (44%) | 26 (39%) | 20 (53%) |  |
| **Fist sign** |  |  |  | 0.24 |
| Yes | 26 (25%) | 14 (21%) | 12 (32%) |  |
| No | 78 (75%) | 52 (79%) | 26 (68%) |  |
| **Butterfly Sign** |  |  |  | 0.35 |
| Yes | 12 (11.5%) | 6 (9%) | 6 (16%) |  |
| No | 92 (88.5%) | 60 (91%) | 32 (84%) |  |
| **Purfling Sign** |  |  |  | 0.82 |
| Yes | 18 (17%) | 11 (17%) | 7 (18%) |  |
| No | 86 (83%) | 55 (83%) | 31 (82%) |  |

Note: *PCNSL*, Primary Central Nervous System Lymphoma; *DEL*, Double-Expressor Lymphoma; *GCB*, Germinal Center B-cell-like. Categorical variables are presented as number of patients (percentage). Continuous variables are presented as Mean ± SD. Categorical variables followed by "(Yes)" indicate that the data in that row represent the number and percentage of cases that meet the condition (i.e., are positive). P-values in bold indicate statistical significance (*P* < 0.05).

**Table S4.** Training and test cohort comparability of baseline characteristics for the GCB vs non-GCB cases

| **Clinical information**  **and MRI features** | **Total GCB+non-GCB**  **(n = 143)** | **Training cohort**  **(n = 91)** | **Test cohort (n = 52)** | ***P* value** |
| --- | --- | --- | --- | --- |
| **Age (years)** | 56.3 ± 13.8 | 55.7 ± 14.62; | 57.4 ± 12.3 | 0.59 |
| **Gender** |  |  |  | 0.32 |
| Male | 82 (57%) | 55 (60%) | 27 (52%) |  |
| Female | 61 (43%) | 36 (40%) | 25 (48%) |  |
| **Tumor Volume (mL)** | 18.2 ± 21.7 | 19.9 ± 24.2 | 15.3 ± 16.3 | 0.055 |
| **Edema Volume (mL)** | 51.4 ± 46.2 | 51.5 ± 43.2 | 51.2 ± 51.5 | 0.75 |
| **Location** |  |  |  | 0.37 |
| Midline | 48 (344%) | 33 (36%) | 15 (29%) |  |
| Hemisphere | 95 (66%) | 58 (64%) | 37 (71%) |  |
| **Tentorial**  **Compartment** |  |  |  | 0.65 |
| Supratentorial | 107 (75%) | 70 (77%) | 37 (71%) |  |
| Infratentorial | 15 (11%) | 8 (9%) | 7 (14%) |  |
| Both | 21 (15%) | 13 (14%) | 8 (15%) |  |
| **Deep Involvement** |  |  |  | 0.64 |
| Yes | 113 (79%) | 73 (80%) | 40 (77%) |  |
| No | 30 (21%) | 18 (20%) | 12 (23%) |  |
| **Multiple Lesions** |  |  |  | 0.92 |
| Yes | 70 (49%) | 43 (47%) | 27 (52%) |  |
| No | 73 (51%) | 48 (53%) | 25 (48%) |  |
| **Necrosis** |  |  |  | 0.44 |
| Yes | 70 (49%) | 50 (55%) | 20 (38.5%) |  |
| No | 73 (51%) | 41 (45%) | 32 (61.5%) |  |
| **Tumor Margin** |  |  |  | 0.16 |
| Regular | 42 (29%) | 23 (25%) | 19 (36.5%) |  |
| Irregular | 101 (71%) | 68 (75%) | 33 (63.5%) |  |
| **Enhancement Pattern** |  |  |  | 0.42 |
| Nonhomogeneous | 94 (66.%) | 62 (68%) | 32 (61.5%) |  |
| Homogeneous | 49 (34%) | 29 (32%) | 20 (38.5%) |  |
| **Midline Shift** |  |  |  | 0.63 |
| Yes | 56 (39%) | 37 (41%) | 19 (36.5%) |  |
| No | 87 (61%) | 54 (59%) | 33 (63.5%) |  |
| **Angular Sign** |  |  |  | 0.28 |
| Yes | 55 (38.5%) | 38 (42%) | 17 (33%) |  |
| No | 88 (61.5%) | 53 (58%) | 35 (67%) |  |
| **Umbilication Sign** |  |  |  | 0.14 |
| Yes | 83 (58%) | 57 (63%) | 26 (50%) |  |
| No | 60 (42%) | 34 (37%) | 26 (50%) |  |
| **Fist Sign** |  |  |  | 0.27 |
| Yes | 38 (27%) | 27 (30%) | 11 (21%) |  |
| No | 105 (73%) | 64 (70%) | 41 (79%) |  |
| **Butterfly Sign** |  |  |  | 0.54 |
| Yes | 12 (8%) | 9 (10%) | 3 (6%) |  |
| No | 131 (92%) | 82 (90%) | 49 (94%) |  |
| **Purfling Sign** |  |  |  | 0.64 |
| Yes | 19 (13%) | 13 (14%) | 6 (11.5%) |  |
| No | 124 (87%) | 78 (86%) | 46 (88.5%) |  |

Note: *PCNSL*, Primary Central Nervous System Lymphoma; *DEL*, Double-Expression Lymphoma; *GCB*, Germinal Center B-cell-like. Categorical variables are presented as number of patients (percentage). Continuous variables are presented as Mean ± SD. Categorical variables followed by "(Yes)" indicate that the data in that row represent the number and percentage of cases that meet the condition (i.e., are positive). *P*-values in bold indicate statistical significance (*P* < 0.05).

**Table S5.** Univariate and multivariate analysis for DEL and non-DEL Groups (Training cohort, n = 66).

| **Characteristic** | **Univariate Analysis** | | **Multivariate Analysis** | | |
| --- | --- | --- | --- | --- | --- |
|  | *OR* (95% *CI*) | *P* Value | *β* Coefficient | *OR* (95% CI) | *P* Value |
| **Age (years)** | 1.02 (0.98, 1.06) | 0.33 |  |  |  |
| **Gender** |  |  |  |  |  |
| Female | Reference |  |  |  |  |
| Male | 0.64 (0.24, 1.70) | 0.37 |  |  |  |
| **Tumor Volume (mL)** | 1.03 (0.99, 1.08) | 0.10 |  |  |  |
| **Edema Volume (mL)** | 1.02 (1.00, 1.03) | 0.03 | 0.01 | 1.01 (1.00, 1.03) | 0.12 |
| **Location** |  |  |  |  |  |
| Hemisphere | Reference |  |  |  |  |
| Midline | 1.16 (0.38, 3.56) | 0.79 |  |  |  |
| **Tentorial Compartment** |  |  |  |  |  |
| Supratentorial | Reference |  |  |  |  |
| Infratentorial | 0.11 (0.01, 1.01) | 0.051 | -1.71 | 0.18 (0.02, 1.74) | 0.14 |
| Both | 1.69 (0.30, 9.56) | 0.55 | 0.61 | 1.84 (0.30, 11.08) | 0.51 |
| **Deep Involvement** |  |  |  |  |  |
| No | Reference |  |  |  |  |
| Yes | 0.58 (0.16, 2.16) | 0.42 |  |  |  |
| **Multiple Lesions** |  |  |  |  |  |
| No | Reference |  |  |  |  |
| Yes | 1.45 (0.55, 3.84) | 0.46 |  |  |  |
| **Necrosis** |  |  |  |  |  |
| No | Reference |  |  |  |  |
| Yes | 1.73 (0.64, 4.64) | 0.28 |  |  |  |
| **Tumor Margins** |  |  |  |  |  |
| Regular | Reference |  |  |  |  |
| Irregular | 1.22 (0.42, 3.54) | 0.72 |  |  |  |
| **Enhancement Pattern** |  |  |  |  |  |
| Homogeneous | Reference |  |  |  |  |
| Non-homogeneous | 0.90 (0.31, 2.64) | 0.5 |  |  |  |
| Angular Sign |  |  |  |  |  |
| No | Reference |  |  |  |  |
| Yes | 0.73 (0.27, 1.95) | 0.53 |  |  |  |
| **Umbilication Sign** |  |  |  |  |  |
| No | Reference |  |  |  |  |
| Yes | 0.53 (0.19, 1.47) | 0.22 |  |  |  |
| Fist Sign |  |  |  |  |  |
| No | Reference |  |  |  |  |
| Yes | 2.31 (0.64, 8.33) | 0.20 |  |  |  |
| **Butterfly Sign** |  |  |  |  |  |
| No | Reference |  |  |  |  |
| Yes | 1.64 (0.28, 9.63) | 0.59 |  |  |  |
| **Purfling Sign** |  |  |  |  |  |
| No | Reference |  |  |  |  |
| Yes | 2.39 (0.57, 9.98) | 0.23 |  |  |  |

Note: *PCNSL*, Primary Central Nervous System Lymphoma; *DEL*, Double-Expressor Lymphoma; *GCB*, Germinal Center B-cell-like. Categorical variables are presented as number of patients (percentage). Continuous variables are presented as Mean ± SD. Categorical variables followed by "(Yes)" indicate that the data in that row represent the number and percentage of cases that meet the condition (i.e., are positive). P-values in bold indicate statistical significance (*P* < 0.05).

**Table S6.** Inter-reader agreement and agreement between manual and automated segmentation for enhancing tumor core and peritumoral edema.

| **Comparison** | **Label** | **DSC, mean ± SD** | **DSC 95% CI** | **Volume ICC** | **ICC 95% CI** |
| --- | --- | --- | --- | --- | --- |
| Reader 1 vs Reader 2 | Enhancing tumor | 0.97 ± 0.05 | 0.89–0.95 | 0.99 | 0.98–0.99 |
| Reader 1 vs Reader 2 | Peritumoral edema | 0.91 ± 0.08 | 0.80–0.88 | 0.98 | 0.97–0.99 |
| Reader 1 vs automatic segmentation | Enhancing tumor | 0.90 ± 0.13 | 0.86–0.92 | 0.99 | 0.98–0.99 |
| Reader 1 vs automatic segmentation | Peritumoral edema | 0.72 ± 0.13 | 0.69–0.75 | 0.97 | 0.95–0.98 |

DSC, Dice similarity coefficient; ICC, intraclass correlation coefficient; SD, standard deviation; CI, confidence interval. DSC values are presented as mean ± SD across cases. The 95% CIs for DSC and volume ICC were estimated using nonparametric bootstrap resampling. Volume ICC was calculated to assess agreement in segmented volume between two annotations.

**Table S7.** Performance of six machine-learning classifiers for distinguishing DEL from non-DEL in the training set (n=66).

| **Model** | **AUC** | **Accuracy** | **Sensitivity** | **Specificity** | **PPV** | **NPV** | **F1 score** |
| --- | --- | --- | --- | --- | --- | --- | --- |
| SVM | 0.862 | 0.791 | 0.838 | 0.733 | 0.800 | 0.780 | 0.819 |
| SGD | 0.847 | 0.806 | 0.865 | 0.733 | 0.805 | 0.810 | 0.834 |
| KNN | 0.892 | 0.791 | 0.784 | 0.800 | 0.833 | 0.744 | 0.808 |
| RF | 0.797 | 0.731 | 0.784 | 0.667 | 0.750 | 0.708 | 0.767 |
| XGBoost | 0.826 | 0.716 | 0.757 | 0.667 | 0.744 | 0.683 | 0.750 |
| LightGBM | 0.848 | 0.791 | 0.784 | 0.800 | 0.833 | 0.744 | 0.808 |

**Table S8.** Performance of six machine-learning classifiers for distinguishing GCB from non-GCB subtype in the training set (n=91).

| **Model** | **AUC** | **Accuracy** | **Sensitivity** | **Specificity** | **PPV** | **NPV** | **F1 score** |
| --- | --- | --- | --- | --- | --- | --- | --- |
| SVM | 0.907 | 0.901 | 0.884 | 0.917 | 0.905 | 0.898 | 0.894 |
| SGD | 0.882 | 0.835 | 0.791 | 0.875 | 0.850 | 0.824 | 0.819 |
| KNN | 0.895 | 0.879 | 0.814 | 0.938 | 0.921 | 0.849 | 0.864 |
| RF | 0.914 | 0.923 | 0.884 | 0.958 | 0.950 | 0.902 | 0.916 |
| XGBoost | 0.953 | 0.813 | 0.744 | 0.875 | 0.842 | 0.792 | 0.790 |
| LightGBM | 0.909 | 0.846 | 0.767 | 0.917 | 0.892 | 0.815 | 0.825 |

Note: GCB, germinal center B-cell-like; SVM, support vector machine; SGD, stochastic gradient descent, implemented as an L2-regularized logistic classifier; KNN, k-nearest neighbors; RF, random forest; XGBoost, extreme gradient boosting; GBM, gradient boosting machine; AUC, area under the receiver operating characteristic curve; Acc, accuracy; Sen, sensitivity; Spe, specificity; PPV, positive predictive value; NPV, negative predictive value; F1 score, harmonic mean of precision and sensitivity. GCB was considered the positive class.


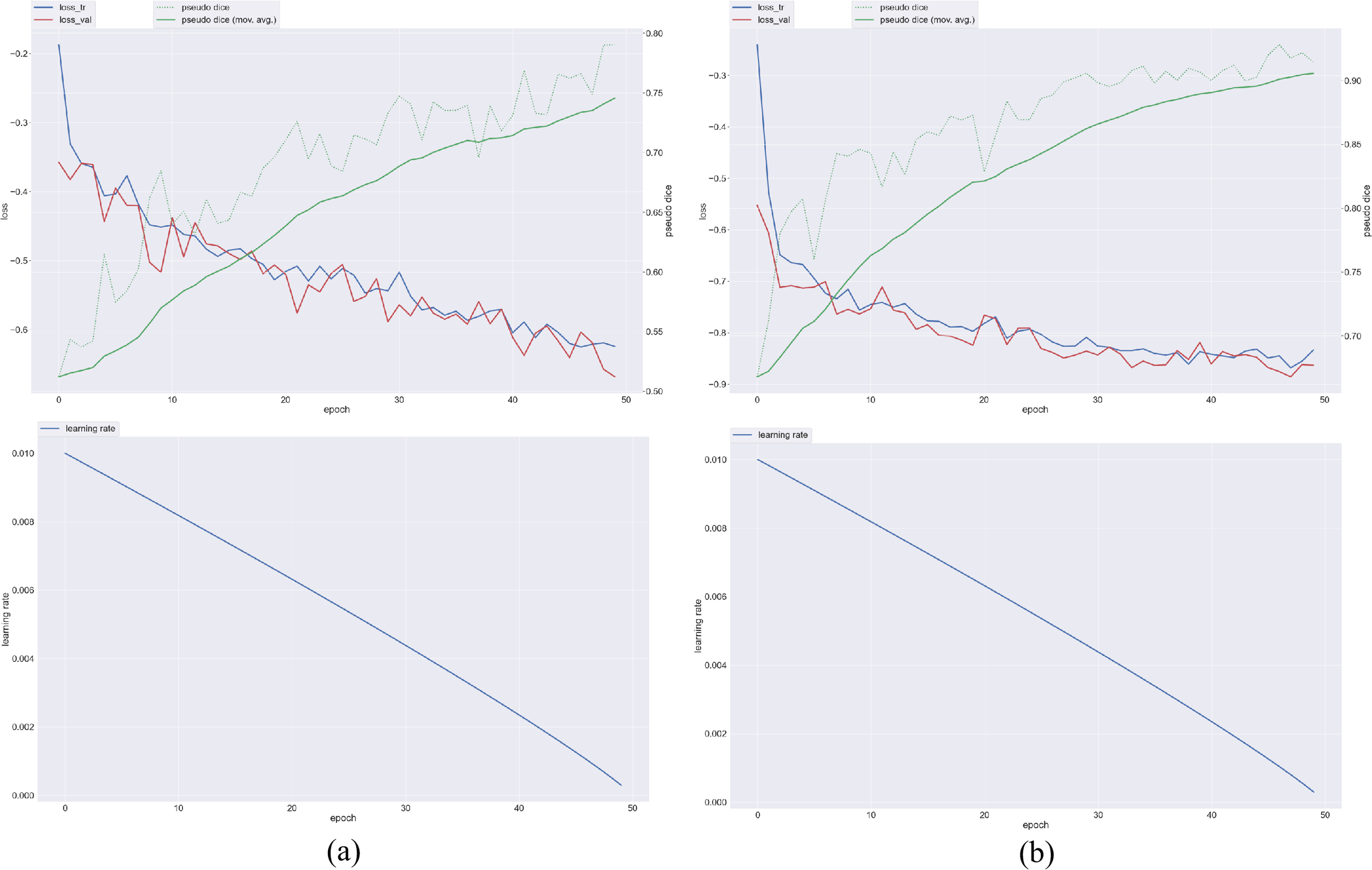


**Figure S1.** Training and validation performance of the nnU-Net v2 automatic segmentation model. (a) Model training metrics for enhancing tumor core segmentation, which achieved a mean validation dice similarity coefficient (DSC) of 0.90. (b) Corresponding training metrics for the peritumoral edema segmentation, which achieved a mean validation DSC of 0.72.


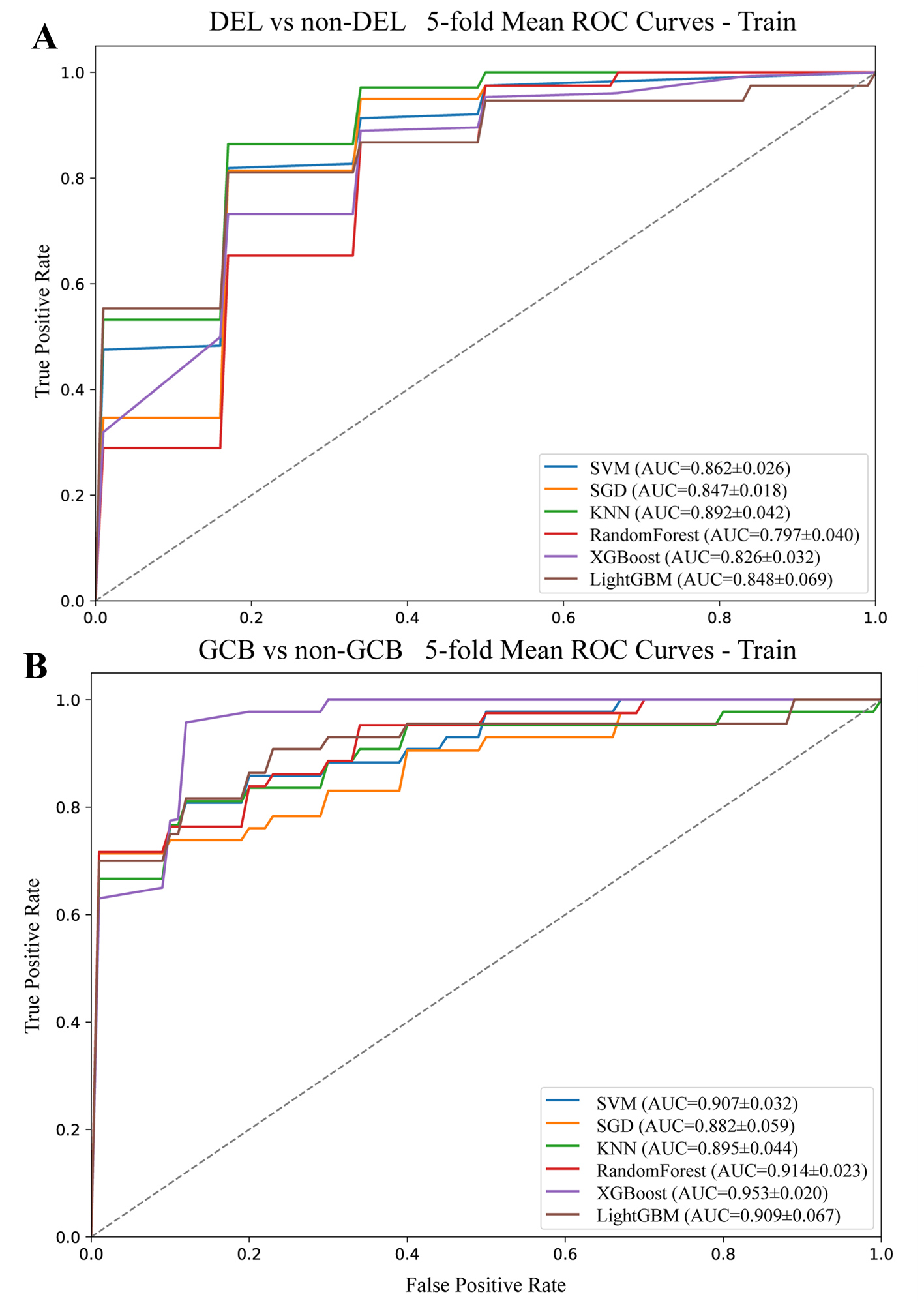


**Figure S2.** (A) ROC curves of six machine-learning classifiers for DEL versus non-DEL classification in the training set. (B) ROC curves of six classifiers for GCB versus non-GCB classification in the training set.
